# Hématome spontané du méso de l'angle colique droit et du colon transverse compliquant un traitement par anti vitamine K: à propos d'un cas et revue de la littérature

**DOI:** 10.11604/pamj.2016.23.52.8231

**Published:** 2016-02-29

**Authors:** Ibrahim Alain Traoré, Cyprien Zaré, Sié Drissa Barro, Ismaël Guibla

**Affiliations:** 1 Service d'Anesthésie-réanimation, CHU Souro-Sanou de Bobo-Dioulasso, Bobo-Dioulasso, Burkina Faso; 2 Service de Chirurgie, CHU Souro-Sanou de Bobo-Dioulasso, Bobo-Dioulasso, Burkina Faso

**Keywords:** Hématome, anticoagulant, hémopéritoine, Hematoma, anticoagulant, hemoperiteum

## Abstract

L'hématome spontané du méso de l'angle colique droit et du transverse est une complication rare du traitement anticoagulant par antivitamine K. Nous rapportons un cas d'hématome spontané du méso de l'angle colique droit et du transverse associé à un hémopéritoine de grande abondance chez un patient traité par antivitamine K pour embolie pulmonaire consécutive à une fracture des plateaux tibiaux droits. Le diagnostic doit être fait en urgence. L’échographie abdominale et la tomodensitométrie confirment le diagnostic. Le traitement non opératoire est la règle. Le traitement chirurgical est indiqué en cas de complications telles que la rupture de l'hématome.

## Abstract (en)

Spontaneous hematoma of transverse mesocolon is a rare complication of anticoagulant treatment with vitamin K. We report the case of spontaneous hematoma of right angle of the transverse mesocolon associated with a hemoperitoneum in a 32-year-old patient treated by fluindione for pulmonary embolism. The diagnosis must be made urgently. The abdominal ultrasound and the scanning confirm the diagnosis. It is important to note that surgery is indicated only in the case of complications such as the risk of rupture of the hematoma.

## Introduction

La prise en charge de la maladie thromboembolique veineuse est basée sur une anti coagulation efficace. En dépit de la standardisation de la surveillance biologique et d'une meilleure définition des objectifs thérapeutiques, le traitement anticoagulant est encore associé à un pourcentage non négligeable d'accidents hémorragiques [1]. Parmi ces complications, on retrouve les hématomes coliques spontanés qui sont exceptionnels mais engagent le pronostic vital [2]. Nous rapportons un cas d'hématome du méso de l'angle colique droit et du colon transverse associé à un hémopéritoine de grande abondance chez une patiente traitée par anti vitamine K pour embolie pulmonaire.

## Patient et observation

Il s'agit d'une patiente de 35 ans, sans antécédent pathologique particulier connu, admise en réanimation pour suspicion d'embolie pulmonaire. Elle avait été admise aux urgences chirurgicales 20 jours plus tôt, pour fracture isolée des plateaux tibiaux droits traitée par un plâtre circulaire cruro-pédieux, sans anticoagulation préventive. L’évolution a été marquée deux semaines plus tard, par la survenue de douleur du membre associée à une tuméfaction ayant motivé l'ablation du plâtre. Une immobilisation par attelle a été alors réalisée. La patiente n'a pas non plus été mise sous anticoagulant. Une semaine plus tard, devant l'apparition de douleurs basithoraciques droites d'intensité croissante accompagnées de dyspnée et de fièvre, elle a été hospitalisée dans une structure privée pendant deux jours avant d’être transférée en réanimation après un traitement à base de paracétamol, métamisole et ceftriaxone. A l'entrée en réanimation, elle a bénéficié d'une oxygénothérapie, une anticoagulation à dose curative (Enoxaparine 4000 UI 2 fois /jour), une analgésie (Paracétamol et Tramadol) et une antibiothérapie (Amoxicilline-Acide clavulanique). Le bilan à l'admission a permis de suspecter une embolie pulmonaire devant: une élévation des D-Dimères (3547,94 ng/ml), une opacité dense homogène sans bronchogramme aérien occupant le tiers inferieur du champ pulmonaire droit à la radiographie pulmonaire et un aspect S1Q3T3 à l'ECG. L'angioscanner thoracique réalisé au deuxième jour d'hospitalisation a confirmé l'embolie pulmonaire. L’échodoppler veineux du membre inférieur droit a mis en évidence une thrombose veineuse profonde de la fémorale superficielle.

**Figure 1:**
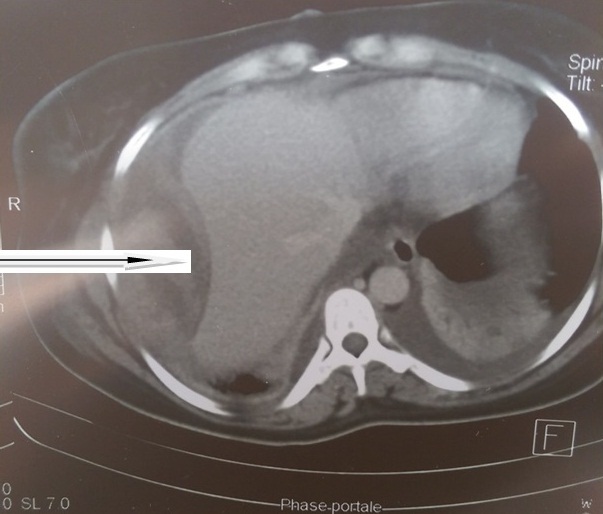
Scannographie abdominale montrant un volumineux hématome extracapsulaire du foie (flèche) avec un hémopéritoine

Le diagnostic d'embolie pulmonaire compliquant une thrombophlébite du membre pelvien droit a alors été retenue. L'héparinothérapie avec l'enoxaparine a été poursuivie associée au Fluindione 20 mg/jour. L'arrêt de l'enoxaparine est décidé au cinquième jour de l'association enoxaparine-fluindione (INR à 2,10). Au sixième jour d'anticoagulation par le Fluindione seul, il est apparu une douleur à l'hypocondre droit à type de pesanteur inhibant l'inspiration profonde. Une échographie abdominale réalisée a objectivé un important hématome extra capsulaire du foie. La patiente a présenté un état de choc hémorragique au septième jour. A l'hémogramme on retrouvait une déglobulisation (hémoglobine à 8,4 g/dL, contre 11, 6 g/dL la veille), le bilan de la coagulation était normal (INR à 2,84). Un remplissage vasculaire a été fait (500 mL de macromolécule) associé à une transfusion sanguine (deux poches de concentré de globule rouge) et de 3 poches de plasma frais congelé (PFC). L'arrêt de la fluindione a aussi été décidé. Devant la persistance de l'anémie malgré une transfusion itérative (6 poches de concentré de globule rouge en cinq jours), une tomodensitométrie (TDM) abdominale réalisée (Figure 1) a objectivé un énorme hématome extracapsulaire du foie et un hémopéritoine de grande abondance. La laparotomie réalisée 48 heures après le diagnostic, a mis en évidence un hémopéritoine de 2,5 L, un important hématome du méso de l'angle colique droit et du colon transverse de 16 cm de grand axe. Les suites opératoires ont été simples.

## Discussion

Les hémorragies digestives constituent la complication la plus fréquente des anti vitamine K (AVK). En effet, les résultats de l’étude ISCOAT (Italian Study on complications of oral anticoagulant) ont montré un taux de 30,4% [3]. Une autre étude menée en Allemagne, a rapporté un taux à 36,7% [3–5]. L'hématome intramural de l'intestin avec hémopéritoine est une complication rare du traitement par AVK. Il correspond à une infiltration hématique segmentaire de l'intestin sur 10 à 40 cm de long, pouvant faire disparaître la lumière intestinale avec hémopéritoine et éventuelle hémorragie digestive. Sa topographie préférentielle est proximale, duodénale, et surtout jéjunale. Les localisations coliques, particulièrement sigmoïdiennes, sont exceptionnelles [2, 6]. La présence d'une douleur abdominale en dehors de tout traumatisme et d'une obstruction intestinale (généralement partielle) peut orienter le diagnostic. L'INR est souvent augmenté au-delà des marges thérapeutiques. Notre patiente a présenté en dehors de tout traumatisme, un hématome du méso de l'angle colique droit et du colon transverse, associé à une hémorragie intramurale et intrapéritonéale. Le traitement anticoagulant semble en être la cause malgré un INR à 2,84. En effet, il a été décrit des cas d'hématomes intramuraux avec un INR légèrement augmenté ou même normal [2]. Si un accident hémorragique peut survenir chez un patient ayant un INR dans la zone thérapeutique attendue, comme ce fut le cas chez notre patiente, le risque de saignement est surtout lié à l'intensité de l'anticoagulation [7]. Le diagnostic repose sur l’échographie, le scanner et l'endoscopie digestive [2, 6] qui doivent être réalisés en urgence. Notre patiente a bénéficié d'une échographie et d'un scanner qui ont permis de mettre en évidence un hémopéritoine de grande abondance et un hématome extracapsulaire du foie qui s'est avéré être un hématome du méso de l'angle colique droit à la laparotomie. La prise en charge non opératoire est la règle dans les hématomes du colon induits par l'anticoagulation. Cette prise en charge consiste à une aspiration digestive par sonde gastrique en cas d'arrêt des matières et des gaz, une transfusion de sang et de plasma frais congelé et l'administration de faibles doses de Vitamine K1. Le PFC est indiqué dans les situations de choc hémorragique ou en cas d'absence de disponibilité des concentrés de complexe prothrombinique [8]. Le traitement de ces hématomes est d'autant plus complexe que l'indication des anticoagulants est impérative. En effet, Il faut éviter une hypercoagulabilité avec risque de thrombose surtout chez les patients présentant une pathologie cardiovasculaire comme c’était le cas avec notre patiente. L'héparinothérapie intraveineuse est alors indiquée car elle permet une meilleure gestion de l'anticoagulation [9]. Bien que le traitement conservateur soit la règle, une intervention chirurgicale peut être nécessaire. L'indication chirurgicale est le plus souvent motivée par la crainte de rupture de l'hématome, la formation d'abcès, la douleur abdominale incontrôlable et le doute diagnostic [2]. Chez notre patiente, la chirurgie a été motivée par la crainte de rupture de l'hématome.

## Conclusion

L'hématome spontané du méso de l'angle colique droit est une complication rare du traitement par AVK. Il se manifeste le plus souvent par une douleur abdominale siégeant à l'hypocondre droit associée à un syndrome occlusif. L’échographie et le scanner abdominal réalisés en urgence permettent de poser le diagnostic. Le traitement non opératoire est la règle, mais un traitement chirurgical peut être indiqué en cas de risque de rupture de l'hématome.
